# Enrollment and Scientific Update for the Multisite SuperAging Research Initiative

**DOI:** 10.1002/alz70857_104075

**Published:** 2025-12-25

**Authors:** Amanda Cook Maher, Robert Bartha, Ivan Culum, Elizabeth Finger, Changiz Geula, Felicia C. Goldstein, Matthew J Huentelman, Andrew Lim, Adam Martersteck, Marsel Mesulam, Ozioma C. Okonkwo, Henry L Paulson, Angela C. Roberts, Edna Rose, Phyllis Timpo, Antoine R Trammell, Richard H. Swartz, Karen Van Ooteghem, Emily J Rogalski, SuperAging Research Initiative

**Affiliations:** ^1^ Michigan Alzheimer's Disease Research Center, Ann Arbor, MI, USA; ^2^ University of Michigan, Ann Arbor, MI, USA; ^3^ Western University, London, ON, Canada; ^4^ Robarts Research Institute, Western University, London, ON, Canada; ^5^ Canadian Centre for Activity and Aging, London, ON, Canada; ^6^ Lawson Health Research Institutes, London, ON, Canada; ^7^ Northwestern ADC Neuropathology Core, Northwestern University Feinberg School of Medicine, Chicago, IL, USA; ^8^ Mesulam Center for Cognitive Neurology & Alzheimer's Disease, Chicago, IL, USA; ^9^ Emory University School of Medicine, Atlanta, GA, USA; ^10^ Neurogenomics Division, Translational Genomics Research Institute, Phoenix, AZ, USA; ^11^ Sunnybrook Research Institute, Toronto, ON, Canada; ^12^ University of Chicago, Chicago, IL, USA; ^13^ Northwestern University Feinberg School of Medicine, Chicago, IL, USA; ^14^ University of Wisconsin School of Medicine and Public Health, Madison, WI, USA; ^15^ Emory University Goizueta Alzheimer's Disease Research Center, Atlanta, GA, USA; ^16^ Sunnybrook Health Sciences Centre, Toronto, ON, Canada; ^17^ University of Waterloo, Waterloo, ON, Canada; ^18^ Healthy Aging & Alzheimer's Research Care (HAARC) Center, Healthy Aging & Alzheimer's Research Care (HAARC) Center, The University of Chicago, Chicago, IL, USA

## Abstract

**Background:**

Established in 2021, the SuperAging Research Initiative is a multisite, longitudinal study focused on identifying resilience and resistance factors that promote successful cognitive aging. “SuperAgers” are defined as individuals age 80+ with episodic memory performance that is average or better for individuals 20‐30 years younger. The SuperAging Research Initiative aims to advance knowledge of the neurobiology of brain aging, resilience, and resistance against “typical” age‐related cognitive decline and pathologic declines seen in Alzheimer's disease and related disorders. The SuperAging Research Initiative is focused on increasing racial‐ethnic, geographical, and educational diversity by enrolling 500+ participants across the United States and Canada. The mid‐project recruitment and enrollment success, baseline participant characteristics, and initial study findings from the unique cohort are highlighted.

**Method:**

Participant enrollment and harmonized data collection is ongoing at five North American sites. The protocol includes behavioral, biological, environmental, genetic, and psychosocial characteristics that may contribute to successful cognitive aging. Two embedded Research Projects provide focused opportunities to extend the depth and breadth of science. Project 1 utilizes state‐of‐the‐art wearable technology to obtain quantitative measurements of daily activity, and Project 2 uses transcriptomic, genetic, and protein profiling to examine immune and inflammatory system parameters.

**Result:**

Across sites, >280 participants (ages 80‐101 with 6‐20 years education) have enrolled in the harmonized protocol using community engaged research (CER) strategies. More than 12 states/provinces are represented. To date, approximately 20% participants identify with a historically underrepresented racial‐ethnic group. Sites leveraging existing CER methodology have enrolled a higher percentage of racially diverse participants (30+%). The Project 1 protocol has shown strong feasibility (>90%), yielding high‐quality data (>95% data recovery) for a fully remote sensor data collection protocol. Project 2 has begun initial analyses, and estimates to date suggest SuperAgers have similar Alzheimer's disease polygenic risk scores compared to their cognitively‐average peers.

**Conclusion:**

The prospective, longitudinal study of SuperAgers is feasible and provides a unique opportunity to identify mechanisms conferring cognitive resilience and resistance against “typical” and pathologic age‐related cognitive decline. Outcomes may identify novel modifiable factors that promote successful cognitive aging.

